# Feasibility and acceptability of advance care planning facilitated by nonphysician clinicians in Japanese primary care: Implementation pilot study

**DOI:** 10.1002/jgf2.586

**Published:** 2022-10-13

**Authors:** Takahiro Mochizuki, Daisuke Yamashita, Chikako Miura, Masakazu Nakamura, Shigeko (Seiko) Izumi

**Affiliations:** ^1^ Japan Association for Development of Community Medicine Practice‐Based Research Network Tokyo Japan; ^2^ Department of Family Medicine Oregon Health & Science University Portland Oregon USA; ^3^ School of Nursing Oregon Health & Science University Portland Oregon USA

**Keywords:** advance care planning, implementation research, nonphysician clinicians, primary care

## Abstract

**Background:**

Implementation of advance care planning (ACP) is urgently needed in Japan, which is one of the most aging countries. This study tested the feasibility and acceptability of ACP facilitated by nonphysician clinicians, and identified barriers and enablers to implementing ACP into Japanese primary care.

**Methods:**

We trained 10 nonphysician clinicians (seven registered nurses, two medical social workers, and one care manager) in four primary care clinics as ACP facilitators. From April to June 2019, the trained facilitators had 19 ACP conversations with their patients. We conducted semistructured interviews and surveys regarding satisfaction and appropriateness of the ACP with patients, family members, ACP facilitators, and primary care physicians (PCPs) regarding their perceptions about ACP facilitated by nonphysician clinicians. Survey data were analyzed using descriptive statistics, and interviews were analyzed using a qualitative content analysis approach.

**Results:**

Majority of patients (75%) and family members (71%) were satisfied with ACP facilitated by nonphysician clinicians. In 71%, ACP facilitators and PCPs thought their ACP facilitation was appropriate and acceptable. Patients stated that they felt comfortable having ACP conversations with nonphysician clinicians. Identified barriers and enablers for ACP included: time restraints, size and organization of the clinics, settings for ACP conversations (ACP at the patient's home), team collaboration, and use of existing system to trigger ACP.

**Conclusions:**

Advance care planning facilitated by nonphysician clinicians was feasible and acceptable in Japanese primary care. Further studies are needed to explore strategies to overcome the barriers and enhance the enablers identified in this study.

## Graphical abstract

We tested the advance care planning (ACP) facilitated by nonphysician clinicians in Japanese primary care clinics. Our findings from the survey and the interviews suggested that it would be acceptable and feasible. In addition, the qualitative data showed some barriers and potential enablers to implement ACP in Japanese primary care settings; time restraints, size and organization of the clinics, settings for ACP conversations (ACP at the patient's home), team collaboration, and use of the existing system to trigger ACP.

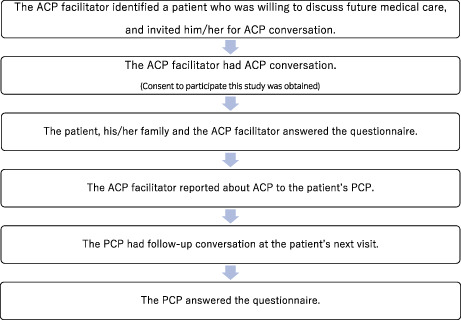

## 1 INTRODUCTION

Advance care planning (ACP) is a process to understand and share a person's values, life goals, and preferences regarding future medical care, and the goal of ACP is to help ensure patients receive medical care that is consistent with their values, goals, and preferences when they are not able to make decisions or express their preferences.^1^ Literature has shown that ACP improves the quality of end‐of‐life care,^2^, ^3^, ^4^ and reduces stress, anxiety, and depression among the family members of the deceased.^5^

Japan is a rapidly aging society, and more than one‐third of the population will be age 65 or older in 2030.^6^ As the population ages, the number of people who live with serious illnesses increases, and so does the need to prepare for future end‐of‐life care. However, the adoption of ACP in Japan has been slow. According to the national survey, only 2.7% of respondents has discussed their end‐of‐life care in detail with family members or healthcare professionals, and more than half have not discussed it at all.^7^ There is a critical need to disseminate and implement ACP broadly in Japan to prepare the aging population to face end‐of‐life decision‐making.

Japanese Ministry of Health, Labour and Welfare developed and started an ACP facilitation training program for registered nurses (RNs) and medical social workers (MSWs) as the main target trainees in 2014.^8^ On the other hand, a study shows a lack of confidence in medical knowledge and ACP facilitation skills among MSWs and care managers (CMs) as a barrier to initiate ACP conversations by these clinicians.^9^ Our internal organization survey shows that physicians tend to think that they should be responsible for ACP and do not recognize the possibility to collaborating with nonphysician ACP facilitators such as ones trained through the program by the Ministry of Health.^10^, ^11^ However, we believed nonphysician clinicians could play key roles to facilitate ACP conversations with patients because of their communication skills and relationship with patients. The purpose of this study is to pilot test the implementation of ACP facilitated by nonphysician clinicians in primary care settings and evaluate its feasibility and acceptability.

## 2 METHODS

### 2.1 Setting, training, and intervention

We invited primary care clinics from the Japan Association for Development of Community Medicine (JADECOM)^12^ to participate in the study. Four clinics from different prefectures, which met the following criteria, agreed to participate in our study. The selection criteria included clinics consisted of 1‐3 physicians, saw an average of 20‐70 primary care patients per day, and had nonphysician clinicians who could participate in our ACP facilitation training. Then, the medical directors of these clinics selected nonphysician clinicians to participate in the ACP training program.

We adapted an existing ACP training program developed by the Japanese Ministry of Health, Labor and Welfare^13^, ^14^ for the primary care setting. Our training program consisted of a 2‐h e‐learning module and a 6‐h in‐person workshop. The e‐learning module included the history, definition, and clinical ethics for ACP. The focus of the workshop was role plays using our ACP facilitation workbook that functions as an ACP conversation guide. ACP facilitation workbook included questions about the patient's goal, the surrogate decision maker, and preferences in their future medical treatments. The trained ACP facilitators identified patients who were willing to discuss future medical care. At the beginning of each ACP conversation, the facilitators explained the objectives of this study and ethical issues such as confidentiality, and the consent was obtained. The ACP facilitators reported to the patient's primary care physicians (PCPs) after an ACP conversation, and the PCPs had follow‐up conversations during the next visit. The study was conducted between April 1 and June 30 in 2019.

### 2.2 Data collection and analysis

**FIGURE 1:**
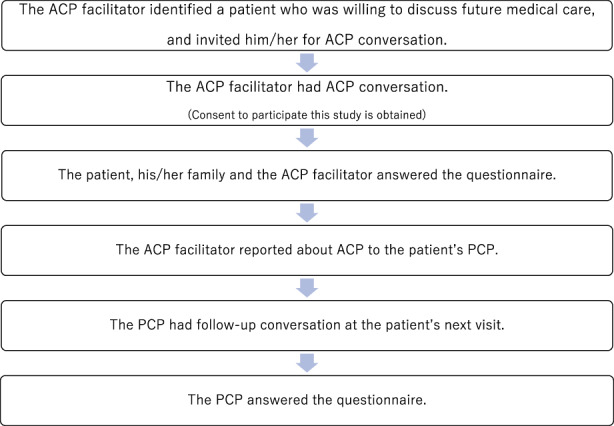
Advance care planning (ACP) workflow. Nonphysician clinicians identified patients and facilitated ACP conversations. Nineteen ACP conversations were conducted between April 1 and June 30 in 2019.

After each ACP conversation, the patient, his/her family member if they were present during the conversation, the ACP facilitator, and the patient's PCP were asked to complete a survey describing their perception about the ACP (Figure 1). Questions included the acceptability of ACP facilitated by nonphysician clinicians and whether patients were able to share their values and preference with family and/or healthcare professionals. The survey data were analyzed using descriptive statistics (Microsoft Excel ver.1808).

In addition, research team members (TM, CM) conducted semi‐structured qualitative interviews with all ACP facilitators, PCPs who worked with ACP facilitators, and patients and their family members who agreed to participate in the interview. Interview questions focused on what worked well, what was challenging, and suggestions to improve ACP conversations and implementation in their practice.

With the participants' permission, all interviews were audio‐recorded and transcribed verbatim for analysis. Three researchers (TM, DY, SI) analyzed the interview data to identify barriers and enablers for ACP in primary care clinics using a directed qualitative content analysis approach^15^ with PARIHS framework^16^: Preliminary and final findings were reviewed and validated by the other research members. Due to the pragmatic nature of this study, we used convenient sampling to recruit participants and data saturation was not sought in this study. This study was approved by the JADECOM Institutional Review Board.

## 3 RESULTS

Seven RNs, one CM, and two MSWs from four primary care clinics (Table 1) participated in the ACP training. All but one trainee were female, and the average years of experience in their role were 21.9 years (Table 2). All PCP participants were male and had 17.8 years of work experience. Three out of ten trained ACP facilitators did not have any ACP conversations and thus did not complete the postconversation survey. A total of 19 patients had ACP conversations between April and June 2019. The average age of patients was 89 (range 79–96), and 12 of them (63.2%) were female. Two‐thirds of the family members who participated in the ACP conversations were patient's adult children, and a third were patient's spouses. Three patients and two family members could not complete the survey mainly due to mild cognitive impairment.

**TABLE 1:** Demographic information of the clinics

	Location	Number of ACP facilitators	Number of physicians	Geriatric care facility	Average number of patients/days
Clinic 1	Kinki region	3	2	w/o	40
Clinic 2	Kinki region	1	2	w/o	30
Clinic 3	Chubu region	1	3	w/	37
Clinic 4	Tohoku region	5	3	w/	71

Abbreviation: ACP, advance care planning; w/, with; w/o, without.

**TABLE 2:** Demographic information of the facilitators, physicians, and patients

Participants	Credential	Gender	*N*
		Experience/Age	
ACP facilitators (*n* = 10)	Registered nurse (*n* = 7)	Female	86 (%)
		Years of experience	24 years (range 17–36)
	Care manager (*n* = 1)	Female	100 (%)
		Years of experience	24 years
	Medical social worker (*n* = 2)	Female	100 (%)
		Years of experience	14 years (range 8–20)
Primary care physicians (*n* = 5)		Female	0 (%)
		Years of experience	18 years (range 11–37)
Patients (*n* = 19)		Female	63 (%)
		Average age	89 years (range 79–96)

### 3.1 Survey

Majority of patients (75%) and family members (71%) who responded to the survey questions were satisfied with the ACP conversations facilitated by nonphysician clinicians, and none was dissatisfied. Seventy‐six percent of patients and all family respondents reported that they could share patients' values and preferences through ACP.

Similarly, 71% of answers from ACP facilitators and PCPs were that the ACP facilitation by nonphysician clinicians was appropriate and acceptable. In open‐ended questions, PCPs reported favorable responses for nonphysicians facilitating ACP because it reduced their burden. On the other hand, ACP facilitators reported that ACP facilitation was time‐consuming for 10 out of 19 ACP conversations (53%), and ACP facilitation was psychologically burdensome for seven out of 19 conversations (32%).

### 3.2 Qualitative interviews

We conducted in‐person qualitative interviews with seven patients with four family members, all 10 ACP facilitators, and five PCPs to identify barriers and enablers to implement ACP. Three ACP facilitators who did not have any ACP conversations were included in the interview to identify what prevented them from having ACP conversations with their patients. Following barriers and enablers were identified and grouped in four domains using PARIHS framework^16^: (1) patient identification (organizational context); (2) ACP program (intervention); (3) characteristics of ACP facilitators (clinician factors); and (4) characteristics of the patient (patient factors) (Table 3).

**TABLE 3:** Barriers and enablers to implement ACP identified from qualitative interviews

Domains	Barriers	Enablers
Patient identification	**Size and organization of the clinics** “Perhaps the biggest problem that our staffs could not have ACP conversations is that the organization is too big. In fact, there is a meeting where doctors, nurses, and MSWs all come together, but it's difficult to get the information across.” (DR) “I know the patients I am in charge of, but I have little information about other patients.” (FA)	**Team collaboration** “I probably could not have done by myself. But since it was just the three of us, we discussed various things and collaborate each other. It created opportunities for other staff members to facilitate ACP.” (FA) “Doctors suggest ACP to patients and their families, and we approach to them. I think this flow is the smoothest. If doctors suggest before, patients would think they should have such conversations.” (FA) **Use of existing system to trigger ACP** “If I have ACP conversations for the reason of updating application form (for long term care service), I think I can approach every time, like ‘How about ACP?’” (FA) “It was at the same time I was preparing the long‐term care service, so I was not worried (to talk about ACP).” (PT) “I also have a thought that this may be the trigger to begin ACP. It's easy to ask when it's a part of a system.” (FA)
ACP program	**Time restraints for ACP** “I tried, but it depends on the other tasks I have to do in the same time frame.” (FA) “It is hard to estimate how long the conversation will take.” (FA) **ACP workbook** “The question, what is your important thing, is the most difficult. Fewer people in their 80s are living with a sense of purpose in life.” (FA) “Those words are a bit difficult. It requires long explanation.” (FA)	**Settings for ACP conversations** “When I had ACP conversations at their home, I could see the backgrounds, and patients were more talkative about their daily lives and values.” (FA) “I think it would be better at home, because patients are relaxed.” (FA) **ACP workbook** “He refused to have the formal ACP conversation meeting, but I knew he should have had some thoughts. I asked him in brief ‘If you become ill, would you like to go to hospital?’ He replied ‘No. My mom had home visits for a long time, and finally died at home. So, if my wife is gonna be well, I want home visits, too.’ I was able to get his brief answer. His wife said ‘We do not discuss such things.’ But (a few key questions about ACP made them that) they started the conversation at home.” (FA)
ACP facilitators	**Psychological burden** “You have to choose words very carefully, especially when you are talking with anxious patients. And that (being very careful what to say) is exhausting.” (FA) “When I asked her what she would do if something happened, she burst into tears and cried. Maybe the feeling when she cared her father and mother came out…The time to ask what to do at the end of life depends on the person, but I was wondering if it was right to ask her.” (FA)	**Nonphysician as ACP facilitator** “Because the nurse knows me well, I think it's good that the nurse is talking with me. I did not mind it was a doctor or a nurse.” (PT) “I think patients talking with us is beneficial. Patients want to be smart and good patients in front of their doctors. They can talk frankly with us, and tell us something they may hesitate to tell doctors.” (FA) “I feel very grateful for that (facilitators had ACP conversations). My role is only supportive. It's quite burdensome to start ACP from scratch, is not it? That's hard to do in my daily work. It's pretty easy if you just check.” (DR)
Patients	**Ability to articulate and disinterest to make a plan** “I do not know what will happen in the future, so I do not think about it.” (PT) **Leave it to family** “Considering what he (the patient's son) has done so far. I'm hoping that he would watch over me until I will enter the grave without telling him anything.” (PT) “Some patients do not tell their true feelings when their children are next to them, because they do not want to bother them.” (FA)	

Abbreviations: ACP, advance care planning; DR, primary care physicians; FA, ACP facilitators; PT, patients.

#### 3.2.1 Patient identification: System for selecting and inviting patients to ACP

##### Size and organization of the clinics

It was hard to identify patients for ACP. In one clinic, only two ACP conversations happened during 3 months study period despite having five trained ACP facilitators. Three ACP facilitators did not have any ACP conversations with their patients. This clinic was a relatively large site attached to a geriatric nursing care facility, and their practice was divided into sections. ACP facilitators' access to patient information was restricted by sections. Each section had a different schedule and workflow; thus, ACP facilitators were dispersed in the multiple sections and were not able to communicate or support each other to implement ACP in their practice workflow. The fragmentation of patient information and more complex practice patterns in a larger organization seemed to be a challenge.

##### Team collaboration

The ACP facilitators and physicians in other sites stated that they took time to have team meetings to discuss how to identify patients for ACP periodically. Sharing information with the care team helped them to identify patients for ACP in a timely manner. It also helped each other to manage the workload to schedule ACP conversations. In addition, they developed a workflow where PCPs introduced ACP to patients first, then warm hand‐off to ACP facilitators that were most successful and well‐received by patients. Forming a shared understanding about ACP and engaging and collaborating with all team members to support ACP was an enabler for successful ACP implementation.

##### Use of existing system to trigger ACP

In Japan, patients need to submit an evaluation form completed and signed by their PCP to the National Health Care Plan to receive long‐term care services such as home care.^6^ A facilitator initiated ACP conversations when patients came to the clinic to obtain PCPs' signatures on this evaluation form. The facilitator stated that it was easy to identify patients when they made an appointment for this evaluation, there was enough time to discuss ACP during this visit, and patients seemed to think it was an appropriate time to talk about future planning. This facilitator felt confident to have ACP conversations with her patients in this context. Other facilitators also stated that using existing system cues such as long‐term care evaluation to flag the patients for ACP was an effective enabler to implement ACP.

#### 3.2.2 ACP program: How to facilitate ACP conversation

##### Time restraints for ACP

Difficulty in securing time to have ACP conversations was the most common barrier identified by the ACP facilitators. The ACP facilitation was added to their routine work and responsibilities. Managing other tasks to create time for ACP conversations was challenging. ACP facilitators stated, “*I tried (to have ACP conversations with my patients), but it depends on the other tasks I have to do in the same time frame*.” “*It is hard to estimate how long the conversation will take. I have to see how patients respond as well as other things I have to do to start the conversation*.”

##### Settings for ACP conversations

Several ACP facilitators expressed difficulties in having ACP conversations in their clinics. As described above, the facilitators found that securing time and fitting ACP conversations in the existing appointment time slot was challenging. Asking a family member to come to the appointment with the patient was another challenge. The working families had to adjust their work schedules to accompany patients and could not stay longer for ACP conversations because they had to go back to work. Knowing that it would add a burden to patients and families, the facilitators were reluctant to schedule an appointment for ACP conversations only.

Four ACP facilitators from two clinics had ACP conversations during their home visits and had positive experiences. The facilitators described that while patients looked uncomfortable talking about ACP in a conference room in the clinic, patients who had ACP conversations at their homes looked relaxed and more open to talk about their daily life and values. The facilitators also stated that seeing the patients in their home environment surrounded by families, pets, home shrines, or hobbies made ACP easier and meaningful to talk about goals and values.

“When I asked, 'what matters most to you?' the patient, who was a wife of a temple priest, told me, 'Since the time I married with him, I cook and serve rice to the Buddha every day. I want to do it until I die. I gave up ringing the temple bell recently (because of the arthritic knee pain). But no matter how painful my knees are, I want to continue to serve rice and pray for the Buddha. That is the most important thing for me.' When I had ACP conversations in the clinic, patients looked uncomfortable. The patients might answer all questions, but they didn’t tell their values in the context of daily life like this patient did.”

Although home visits took a much longer time, including traveling time, the facilitators expressed their satisfaction to have meaningful ACP conversations during home visits. The facilitators and physicians added that ACP during the home visit would be a potential enabler because it was less burdensome for patients, and family members were more likely to join the conversations being nearby.

##### ACP workbook

Our ACP workbook included a series of 19 questions in five steps. Many ACP facilitators expressed difficulties using this ACP workbook. Facilitators said the descriptions and terms were too long and cumbersome, especially for older adults who might have mild cognitive impairment and/or hearing problems. The workbook included some questions about hypothetical end‐of‐life situations, and some patients found it difficult to imagine them. For those who had never thought about goals, values, or preferences of care before, it was difficult to answer these questions at the moment. The facilitators stated that patients needed time to think about each question, and it took a long time to go through all questions in the workbook and was burdensome for patients.

Because of these difficulties, some facilitators modified the approach by limiting the number of questions, using their own terms or local dialect, or embedding a few questions from the workbook in a usual visit instead of setting up a separate ACP visit. The facilitators reported that these approaches worked well because it was easier to have the conversation as part of routine care, patients accepted and responded more, and it was sufficient to let patients and families start ACP.

#### 3.2.3 Characteristics of ACP facilitators

##### Nonphysician clinicians as ACP facilitators

One of the main objectives of this study was to explore the acceptability of nonphysician clinicians as ACP facilitators. Overall, patients were comfortable discussing ACP with the facilitators who were not physicians. One patient stated, “*Because the nurse knows me well, I think it's good that the nurse is talking with me. I didn't mind it was a doctor or a nurse*.” A facilitator stated, “*I think patients talking with us (instead of with doctors) is beneficial. Patients want to be smart and good patients in front of their doctors. They can talk frankly with us, and tell us something they may hesitate to tell doctors*.” PCPs commented that the contents of ACP conversations by the facilitators were appropriate and valuable, and PCPs were appreciative of them because they reduced the workload for physicians.

##### Psychological burden

Facilitators perceived ACP as a sensitive topic, and they were concerned that patients might think they were seriously ill and dying if the facilitators started ACP conversations abruptly. Several facilitators were hesitant to initiate ACP conversation because they felt bad forcing patients to think about death or terminal illness. *“You have to choose words very carefully, especially when you are talking with anxious patients. And that (being very careful what to say) is exhausting.”* Although it was emphasized that the focus of ACP was to learn patient's goals and values rather than end‐of‐life decision‐making in our training, the facilitators still connected ACP with end‐of‐life decision‐making, and the psychological burdens to talk about end‐of‐life care steered them away from starting ACP conversations.

#### 3.2.4 Characteristics of patients

##### Ability to articulate and disinterest in making a plan

One of the common challenges the facilitators described was the patients' inability to articulate their thoughts about values, goals, and preferences for care. Many patients said, “*I don't know what will happen in the future, so I don't think about it,”* or “*You cannot tell what will happen. Life happens, there is nothing I can do*”, and showed disinterest in talking about or making a plan for future care. Some facilitators described that the older generations tended to be not talkative and rarely expressed their feeling or thoughts. Other facilitators also described that many patients had mild cognitive impairment, and their participation in the ACP conversation was limited or not coherent.

##### Leave it to family

Another common response from patients was to leave it to the family. Many patients said that they would leave the end‐of‐life decision‐making to their families because families knew what would be the best for them. The patients assumed that their families knew their values and preference without talking, so they did not feel the need for ACP. However, a few facilitators speculated that some patients might have preferences for care but were not telling them to their families because they did not want to be a burden to their family.

## 4 DISCUSSION

Our findings from the survey and the interviews suggested that ACP facilitation by nonphysician clinicians would be acceptable and feasible. The qualitative data illustrated some barriers and potential enablers to implement ACP in primary care settings.

First, training nonphysician clinicians about ACP facilitation alone was not adequate to implement ACP. The ACP facilitators who worked alone without support from their team struggled to identify patients and manage the time for ACP conversations. Similar to the previous studies, by engaging and sharing the goals of ACP with other team members through team huddle or regular meetings, some clinics were able to develop a workflow to identify patients for ACP and to support facilitators to have time for ACP conversations.^17^, ^18^, ^19^ Collaboration with physicians creating a workflow in which a physician introduces ACP to a patient and warm hand‐off to a facilitator for the conversations was an effective strategy that increased the frequency and acceptance of ACP by patients. It is critical to engage all relevant team members and to build a sustainable system to facilitate ACP conversations, rather than relying on individual facilitator's ability.^20^ Possible strategies to implement and facilitate the adoption of an intervention such as ACP conversations in practice include organizing clinician implementation team meetings, building a coalition, and (re)designing workflow,^21^, ^22^ which would overcome the less confidence of nonphysicians clinicians.^9^ We recommend to include these evidence‐based implementation strategies in the ACP program in addition to the ACP facilitation skill training.

Participants described that having ACP conversations in their clinic was difficult to schedule, burdensome to patients and their families, making them uncomfortable, and not effective to solicit patient's goals and values. Instead, some ACP facilitators suggested that having ACP conversations at patients' home may be a feasible and effective approach. However, not all primary care clinics in Japan do home visits or have those resources. Adapting the ACP conversations to fit the existing workflow based on the current practice and resources, such as home visits, the long‐term care service evaluation visits, or breaking a conversation into a series of questions over the course of multiple visits would be a better systematic and scalable strategy.^23^

The best way to facilitate ACP in the Japanese primary care settings needs further assessment. According to the participants, our ACP program was not easy to conduct. We adapted the ACP training program developed by the Ministry of Health, Labour and Welfare of Japan,^13^ which was mostly based on the concept of ACP in Western countries.^24^ Although we made some modifications in expressions and terms prior to the study based on the input from clinicians, the participants still found our ACP workbook difficult to use because the language was awkward, the questions were difficult for patients to understand or respond to, and it was too long for their patients. Our findings of patients' disinterest in ACP or their attitude to leave healthcare decisions to the family were noteworthy. The involvement of the family member may be more important. The Japan Geriatrics Society announced a statement in 2019, that there was a necessity to promote ACP based on the Japanese context.^25^ Exploring the family perspective along with patients should be emphasized in the training.

### 4.1 Limitations

Limitations of this study include the small convenient sample of participating clinics, facilitators, and patients and families. Another limitation of this study is the lack of data to assess the effectiveness of our ACP training. Given the small sample size, we did not collect data to examine the trainees' competency to facilitate ACP. The responses from some ACP facilitators in the interviews made us question their understanding of the goals and approach for the ACP program. However, despite these limitations, both clinicians and patients were satisfied with the ACP conversations, and we believe our study was valuable. Furthermore, the development of an ACP team training program to foster clinicians' skills to facilitate ACP adapted for Japanese context would be the key to delivering the care that is concordant with a person's goals and values in the rapidly aging society in Japan.^26^

## 5 CONCLUSION

Our study demonstrated the possibility of nonphysician clinicians facilitating ACP in Japanese primary care clinics. Our study also uncovered the potential enablers and barriers to implementing ACP, such as ACP during home visits, team collaboration, and mismatch against the imported concept of ACP. Further studies to identify key elements of culturally appropriate ACP in the existing Japanese healthcare system and education are critically needed.

## FUNDING INFORMATION

This study is supported by Sasakawa Health Foundation (2019A 005).

## CONFLICT OF INTEREST

The authors have stated explicitly that there are no conflicts of interest in connection with this article.

## PATIENT CONSENT FOR PUBLICATION

Obtained.
